# Concrescence: can the teeth involved be moved or separated?

**DOI:** 10.1590/2177-6709.25.1.020-026.oin

**Published:** 2020

**Authors:** Alberto Consolaro, Omar Hadaya, Dario A. Oliveira Miranda, Renata Bianco Consolaro

**Affiliations:** 1 Universidade de São Paulo, Faculdade de Odontologia de Bauru (Bauru/SP, Brazil).; 2 Private practice (Maringá/PR, Brazil).; 3 Universidade Estadual de Feira de Santana, Departamento de Saúde (Feira de Santana/BA, Brazil).; 4 Faculdades Adamantinenses Integradas (Adamantina/SP, Brazil).

**Keywords:** Concrescence, Cement, Third molar, Dental anomaly

## Abstract

The atrophy of the periodontal ligament places the tooth very close to the bone or another tooth, as occurs in unerupted teeth. The absent interdental bone and the lack of functional periodontal stimulus may lead to the fusion of the appositional layers of cement between the roots of the teeth. Concrescence almost always occurs in the region of the maxillary molars. Asymptomatic, it should always be remembered when the proper response to orthodontic movement is not obtained, and there is no apparent explanation. When surgically extracting a tooth and there is resistance, insisting will not be the best strategy. Moving the teeth with concrescence is not convenient, as it requires very intense forces. Once separated, these teeth can be considered normal for movement. It is possible to separate two teeth presenting concrescence, but it depends on the extension of the area, the surgical access and, especially, the clinical convenience. The tooth to be extracted will be repaired with new cement deposited in the sectioned area. The simple separation with the maintenance of the proximity and the lack of function of one of the teeth will cause a new concrescence. After a period of 1 to 3 months, the separated teeth are biologically prepared to be moved. The most important detail in this separation of teeth presenting concrescence is that the diagnosis should be made in advance, and not at the time of the intervention.

## RESUMO

A atrofia do ligamento periodontal coloca o dente muito próximo do osso e/ou de outro dente, como ocorre em dentes não irrompidos. O osso interdentário ausente e a falta de estímulo funcional periodontal podem levar à fusão das camadas aposicionais de cemento entre as raízes dos dentes. A concrescência ocorre quase sempre na região dos molares superiores. Assintomática, ela deve sempre ser lembrada quando não se obtém a resposta adequada a uma movimentação ortodôntica, sem uma explicação aparente. Cirurgicamente, quando se está extraindo o dente e ele oferece resistência, insistir não será a melhor estratégia. Movimentar os dentes com concrescência não é conveniente, pois requer forças muito intensas. Depois de separados, esses dentes podem ser considerados normais para a movimentação. É possível separar dois dentes em concrescência, mas depende da extensão da área, do acesso cirúrgico e, em especial, da conveniência clínica. O dente a ser extraído vai reparar-se, com novo cemento depositado na região seccionada. A simples separação, com manutenção da proximidade e da falta de função de um dos dentes, fará com que uma nova concrescência se estabeleça. Transcorrido o período de 1 a 3 meses, os dentes separados poderão, biologicamente, ser movimentados. O detalhe mais importante nessa separação de dentes com concrescência é que o diagnóstico deve ser feito com antecedência, e não no momento da intervenção.

**Figure 1:**
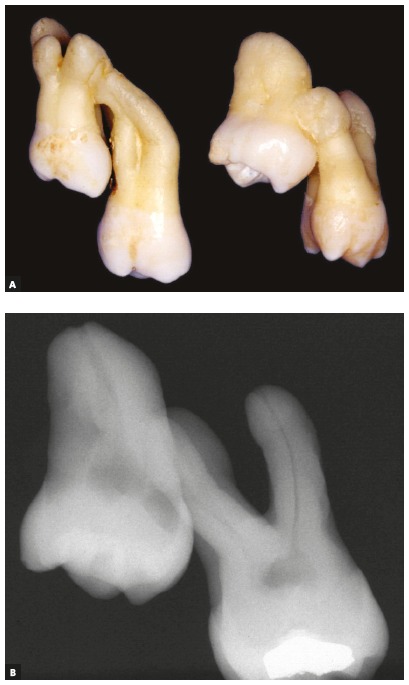
Second and third molars joined by cement, in teeth with and without hypercementosis. Radiographic imaging does not allow root individualization in teeth isolated from other tissues; in vivo, this individualization tends to be even harder.

The union of two teeth by cement is called concrescence^1^ (Fig 1). This is due to the proximity of the roots of two teeth involved, especially when both or one of them is without functional activity of the periodontal tissues. The union of teeth by the dentin and or enamel, besides the cement, characterizes the fusion.

## HOW CONCRESCENCE OCCURS: THE FORMATION MECHANISM

The cement is deposited in successive thin appositional layers that incorporate the periodontal collagen fibers. This process is very slow, but progressive. The active function of the ligament keeps the periodontal space between 0.2 and 0.4 mm thick, thanks to the Malassez Epithelial Remains, which continuously release the EGF mediator, or epidermal or epithelial growth factor, as with all body epithelia. 

There is no part of the body where the epithelium is a direct neighbor to the bone. Always between an epithelium and bone there will be connective tissue intermediating and dissolving the released EGF. EGF in the periodontal ligament stimulates bone resorption on the alveolar surface, which does not occur with the root surface, since cementoblasts do not have receptors for it. The average thickness of the periodontal space is 0.25 mm.

This physiology makes us understand how teeth so close, and often presenting crowding, do not touch the interradicular bone or the root with another root. The maintenance of periodontal space by EGF released from Malassez Epithelial Remnants is really charming, for its subtlety and efficiency.

In non-erupted teeth, alveolodental ankylosis eventually occurs, succeeded by replacement root resorption, and this is explained by excessive atrophy resulting from years without periodontal function. Periodontal ligament atrophy places the tooth very close to the bone, and either bone bridges may form, deviating from the Malassez Epithelial Remnants.

Similarly, when there are two teeth, and one of them is without significant periodontal activity because it is not erupted or is in infraocclusion, over time the cement may approach the root of the other tooth gradually and silently (Figs 1 and 3). The interdental bone may have atrophied equally. This set of absent interdental bone and lack of stimulation of periodontal function can lead to the fusion of appositional layers between two roots of two teeth; or even between two roots of the same tooth.

**Figure 2A:**
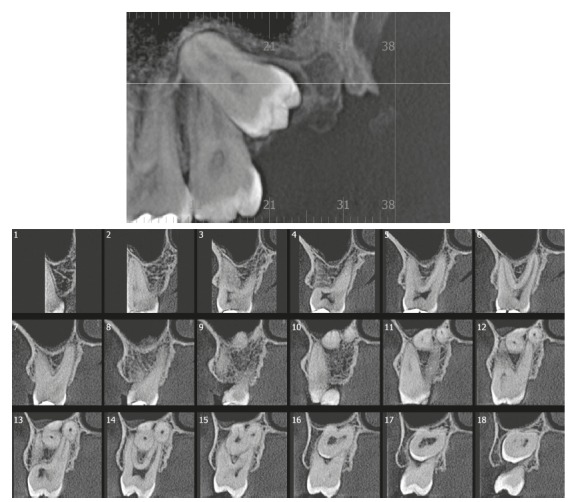
The left third molar was not present in the mouth of this 36-year-old patient, and an imaging evaluation was required. Tooth #38 was joined to tooth #37 by the cement in the palatal (slices 13 to 15) and distal-buccal roots (slices 14 to 16).

**Figure 2B:**
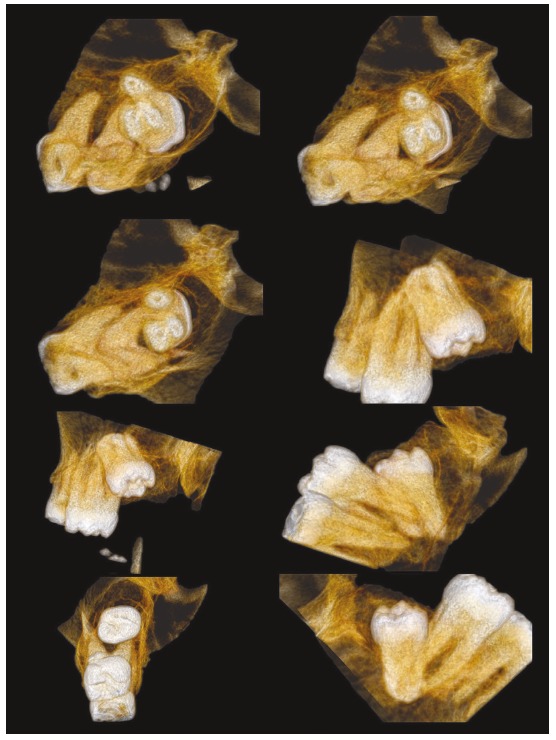
In 3D images of the clinical case of the previous figure, tooth #38 was joined to tooth #37 by the palatal and distal-buccal roots from various angles of observation.

**Figure 2C:**
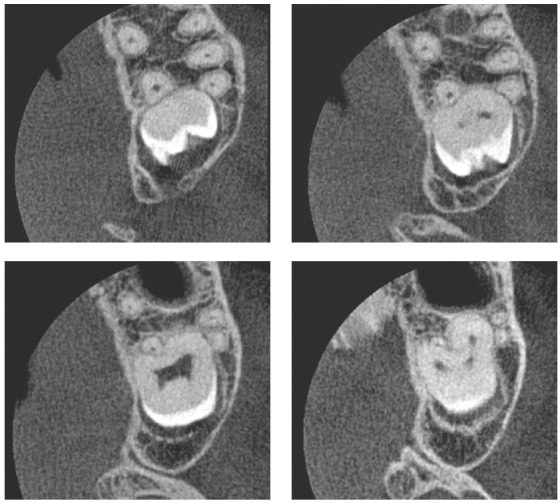
In the axial reconstructions of the clinical case of the previous figure, the union of the palatal and distal-buccal roots of tooth #38 and tooth #37 is even more evident.

**Figure 3:**
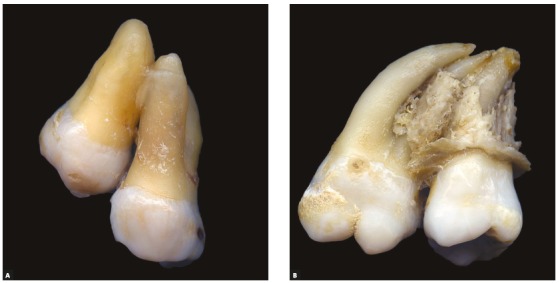
A) Dental specimens obtained after surgical extraction of third molars without previous diagnosis of concrescence with the maxillary second molars, that were extracted at the same time, as well as some bone fragments. In B, maxillary second molar presenting with a discreet hypercementosis.

When the bone meets the tooth, occurs the alveolodental ankylosis. The bone continues to reshape continuously and the tooth will be gradually replaced by bone over months and years. In concrescence, the cement does not undergo remodeling, but presents continued apposition. The meeting of two cements, necessarily and initially, is made by two very thin pre-cement layers that have not yet been mineralized, as well as two “gelled” layers that now fuse and mineralize together (Figs 1 and 3). Thus the dental concrescence is established.

Many years ago, concrescence was classified as "true" when it occurred with developing teeth, and "acquired" concrescence that was established between two teeth that were already fully formed. For practical purposes, if the union is made only by cement, there is no way to differentiate them. If there is also dentin union, as shown in some works, then it is no longer about concrescence but about fusion.

Inflammation due to trauma or microbial contamination when involving teeth tends to cause inflammatory root resorption and even alveolodental ankylosis followed by replacement resorption. The main cause of concrescence is the proximity between two or more teeth due to the lack of space for each one to develop individually.

## CLINICAL ASPECTS TO BE CONSIDERED IN THE DIAGNOSIS AND TREATMENT OF CONCRESCENCE

Concrescence occurs most often in the maxillary molar region, with special emphasis on the involvement of the third molar (Figs 1 and 2), but there are cases involving the first molar.^2^ In this region, the bone space for the teeth is not always sufficient to contain them in the normal position in the dental arch. It is common for the maxillary third molar to turn its crown to the distal, in the tubule of the maxilla, and its root development ends up bringing it too close to the roots of the maxillary second molar, as shown in Figure 2.

Asymptomatic, concrescence should always be remembered when the proper response to orthodontic tooth movement is not readily attained, and there is no apparent explanation for the lack of tooth displacement. There is no direct relationship between concrescence and the occurrence of hypercementosis,^3^ from the statistical and clinical imaging point of view, but in many cases both are present.

In addition to concrescence, ankylosis and replacement resorption should also be remembered when a tooth does not move when expected and without an apparent cause. New images or reexamination of previously obtained images will reveal the accurate diagnosis.

Surgically, likewise, when the tooth is being extracted and it offers resistance, insisting will not be the best strategy, but rather getting a new image and knowing if it has no concrescence, or even ankylosis and replacement resorption. A maxillary third molar, in particular with the second molar, may lead to the loss of the latter in case of persistence during extraction. There are cases of concrescence between a normal tooth and another supernumerary tooth.^4^

Moving teeth presenting concrescence is not convenient, as it requires very intense forces and tends to induce more severe root resorption if dislocation occurs. Once separated, these teeth can be considered normal for movement. Another drawback of orthodontic movement of teeth in concrescence is the fact that they are very likely to act as anchor points in the mechanics used, and to escape professional control.

## IS IT POSSIBLE TO SEPARATE TWO TEETH IN CONCRESCENCE?

Yes, but it depends on the extent of the joined area in this relationship between the two teeth, the surgical access and especially the clinical convenience. Most of the time, the clinical interest is for the extraction of the maxillary third molar, one of the teeth most involved with concrescence. Hardly concrescence involves the apical foramen to the extent of separation requiring prior endodontic treatment. Endodontic treatment is often required due to caries and pulp necrosis.

If they are like the teeth in Figures 1 and 2, it is biologically possible to separate them. The tooth to be moved will be repaired with new cement deposited in the sectioned region. But, before cementoblasts invade and repair this area, during repair - for two to three weeks-, it will undergo imperceptibly surface resorption and will soon be covered by new cement. The simple separation with the maintenance of the proximity and lack of function of one of the teeth will cause a new concrescence to be established, i.e., one of the teeth must be extracted or distanced from the original site via orthodontic movement.

After a repair period of 1 to 3 months has elapsed, the separate teeth are biologically capable of being moved, if this is clinically convenient or desirable. The most important detail in this separation of teeth in concrescence is that the diagnosis must be made in advance, and not at the time of intervention, that is, it requires specific planning for this procedure.

## DIFFERENTIAL DIAGNOSIS OF DENTAL CONCRESCENCE

The differential diagnosis is made with the “fusion”, an anomaly characterized by the union between two teeth by dentin and or enamel, besides the cement. In the case of “tooth gemination” , a single dental germ unsuccessfully attempts to give rise to two teeth, parking in the middle of the process, leaving the involved tooth larger in its mesiodistal size. 

Fusion and gemination almost always affect the anterior teeth, while concrescence involves the second and third maxillary molars. Dental crowns in fusion and gemination are clinically modified, anticipating differentiation with free and morphologically normal clinical crowns.

If the event detected by the clinician is the lack of orthodontic movement, the differential diagnosis of concrescence is made with the alveolodental ankylosis and replacement resorption, as previously explained, and the detailed analysis of the images will allow a safe differentiation.

## FINAL CONSIDERATIONS

In many cases described as concrescence, the teeth involved are also joined by dentin, which characterizes tooth fusion. The diagnostic and conceptual criterion of concrescence requires that the fusion of structures between two or more teeth be done exclusively by cement. 

In clinical cases presented in the anterior region, the proximity of the roots does not mean concrescence, because it requires the loss of root individuality at some points, without contour or continuous well-defined radiolucent or hypodense lines. In CT scans, some areas of concrescence are slightly hypodense, as if they were a rudimentary radiolucent line around the dentin, and this is due to the low mineralization of cement, a tissue with 50% organic part in its composition.

Concrescence in anterior teeth is extremely rare, and even rarer between two teeth in function in the dental arch. Two teeth with active function of the periodontal ligament cannot eliminate the periodontal ligament. In dental trauma, the death of Malassez Epithelial Remnants leads to alveolodental ankylosis and resorption by substitution rather than to concrescence.

The tomography and its beautiful images,^5^ ^,^ ^6^ including 3D, have provided a very accurate diagnostic acuity and allow an accurate diagnosis of dental concrescence based on very precise conceptual and imaging criteria applicable to all specialties of human knowledge.^7^ ^-^ ^10^
